# Adolescents experiencing internalizing symptoms have more favorable attitudes toward online mental health interventions: A cross-sectional study conducted in outpatient services

**DOI:** 10.1192/j.eurpsy.2026.12056

**Published:** 2026-07-31

**Authors:** I. G. Yilmaz-Karaman, E. Toktay Duran, T. Gunduz

**Affiliations:** 1 Psychiatry, Eskisehir Osmangazi University, Eskisehir; 2 Psychiatry, Antalya Training and Research Hospital, Antalya, Türkiye

## Abstract

**Introduction:**

E-mental health interventions are cost-effective and scalable, but their acceptability may vary across clinical profiles. Adolescence is a developmental period when mental health problems emerge and intensify.

**Objectives:**

This study aimed to identify which symptom profiles are associated with more favorable attitudes toward e-mental health interventions.

**Methods:**

In this cross-sectional study, 86 adolescents (70 girls, 16 boys; mean age = 16.0 ± 0.81 years) were recruited from outpatient child and adolescent psychiatry services. Participants completed a Demographic Information Form, the Strengths and Difficulties Questionnaire (SDQ) to assess internalizing and externalizing symptoms, and the E-Therapy Attitudes Measure to gauge perceptions of e-mental health interventions. Associations between symptom profiles and attitudes toward e-mental health interventions were examined using correlation and regression analyses.

**Results:**

Internalizing symptoms correlated with the Perceived Usefulness and Helpfulness subscale of the E-Therapy Attitudes Measure (r = 0.226, p = 0.036), whereas externalizing symptoms showed no association with either subscale (p > 0.05). In univariate logistic regression, gender had no effect on Perceived Usefulness and Helpfulness. Internalizing symptoms significantly predicted higher Perceived Usefulness and Helpfulness toward e-mental health intervention (B = 0.318, t = 2.127, p = 0.036).

**Conclusions:**

Adolescents with higher internalizing symptoms report more favorable perceptions of e-MHIs, while externalizing symptoms and gender are not associated with attitudes. These findings suggest that e-mental health interventions may be particularly acceptable to youth experiencing internalizing difficulties, supporting targeted dissemination and engagement strategies within outpatient services.

**Disclosure of Interest:**

None Declared

